# Clinically relevant behavioral endpoints in a recurrent nitroglycerin migraine model in rats

**DOI:** 10.1186/s10194-016-0624-y

**Published:** 2016-04-19

**Authors:** Kenneth J. Sufka, Stephanie M. Staszko, Ainslee P. Johnson, Morgan E. Davis, Rachel E. Davis, Todd A. Smitherman

**Affiliations:** Department of Psychology, University of Mississippi, Oxford, MS 38677 USA; Research Institute of the Pharmaceutical Sciences, University of Mississippi, Oxford, MS 38677 USA; Department of Pharmacology, University of Mississippi, Oxford, MS 38677 USA

**Keywords:** Migraine, Nitroglycerin, Headache, Translational research, Animal models

## Abstract

**Background:**

This research sought to further validate the rat nitroglycerin (NTG) migraine model by comparing the effects of single versus recurrent NTG episodes on behavioral endpoints that mirror ICHD-3 diagnostic criteria for migraine, and to determine if the altered behavioral endpoints are reduced after administration of sumatriptan.

**Methods:**

Separate cohorts of rats were administered NTG (10 mg/kg/2 ml) or saline (Experiment 1: single injection; Experiment 2: repeated injections; Experiment 3: repeated injections with sumatriptan [0.0, 0.3 and 1.0 mg/kg/ml] rescue. Behavioral endpoints were assessed 2 h after final NTG administration and included time in light/dark chambers for photophobia and activity, pain facial ratings, and cool (5 °C) and warm (46 °C) tail dip.

**Results:**

The first two experiments demonstrated that repeated (*n* = 5) but not single NTG injections produced photophobia, decreased activity, and yielded less weight gain than saline injections. Experiment 3 showed that sumatriptan attenuated hypoactivity, reduced facial expressions of pain, and reversed weight alterations in a dose-dependent manner.

**Conclusions:**

These findings identify numerous clinical homologies of a recurrent NTG rat migraine model that may be useful for screening novel pharmacotherapies.

## Background

Migraine affects 12–16 % of population annually [1, 2] and is the third most common medical condition in the world [3], contributing to significant disability and reduced quality of life [3, 4]. Despite its prevalence and impact, migraine remains undertreated [1] and its pathophysiology incompletely understood. In part these challenges stem from a need for more translational research using well-established animal simulations [5], in which homologies are demonstrated between the human clinical presentation and animals’ behavioral responses. Although rodents have been used in pain research for well over six decades, work to validate a migraine simulation is a comparatively recent undertaking. Although no single model is likely to explain all aspects of migraine [6], animal simulations have already informed our understanding of the genetic basis of familial hemiplegic migraine and the role of cortical spreading depression in aura [7, 8].

Existing animal models of migraine vary considerably in their methods of inducing migraine. Two common approaches include peripheral administration of nitroglycerin (NTG) [9] or intracranial administration of inflammatory mediators [10, 11]. NTG administration produces attacks phenotypically similar to spontaneous migraine attacks [8, 12] and sensitizes trigeminal and cortical structures that underlie migraine pain [13]. Most rodent NTG models use a single injection, but in humans migraine presents as disorder involving recurrent episodic manifestations [14]. One recent study has shown that a model using repeated NTG injections holds promise for studying the ontogeny of migraine as a chronic disorder [15].

Rodent migraine models often use endpoints that include thermal or tactile allodynia as indicators of migraine-related sensory hypersensitivity [7, 10, 16]. Allodynia is reported by nearly 2/3 of migraineurs [17] but is conceptualized primarily as a marker of migraine progression [18, 19], infrequently assessed clinically [20], and not a diagnostic criterion [14]. Thus, existing rodent models may not adequately map onto the human clinical presentation as they rarely quantify spontaneously emitted pain behaviors [21, 22], which comprise patients’ primary reason for seeking treatment.

The aims of the present research thus were to further validate the NTG migraine model in rats by comparing the effects of single versus multiple NTG episodes on behavioral endpoints that reflect ICHD-3 diagnostic symptoms of migraine, including pain intensity, photophobia, and alterations in motor activity. Changes in body weight and thermal allodynia were also assessed. An additional validation step was to determine if NTG-sensitive endpoints were reduced after administration of sumatriptan.

## Methods

### Ethics, consent, and permissions

Housing and experimental procedures were approved by the university’s IACUC (protocol #13–023).

### Subjects and housing characteristics

Separate cohorts of naïve male Sprague–Dawley rats (Harlan Laboratories; Indianapolis, IN, USA) weighing 250–330 g served as subjects for each of three experiments; animals were randomized to treatment condition. Rats were pair-housed in 13 × 21 × 22 cm polyethylene tubs. Food (Teklad 7001, Teklad Diets, Madison, WI, USA) and water were available *ad libitum*. Room temperature was maintained at 22 +/− 4 ° C, and overhead florescent illumination was maintained on a 12/12-h cycle (lights on at 07:00). Testing procedures were carried out during the middle third of the light cycle.

Experiments 2 and 3 involved repeated NTG-migraine episodes over a 2-week period with testing following the last episode. Small igloo-type enclosures (PVC end caps 16.5 cm × 7.5 cm with painted black interior and arched opening cut into the sidewall) were placed in the home cages to permit rats to reduce exposure to colony room lighting after NTG injections.

### Drugs

Nitroglycerin (NTG; SDM®27, Copperhead Chemical, Tamaqua, PA, USA) was diluted in a 50:50 propylene glycol and ethanol vehicle. NTG dose was 10 mg/kg based on previous studies that this dose reliably produced alterations in allodynia 2 h after administration [15, 16]. To avoid the possibility of migraine-related activity of ethanol in the vehicle, saline was administered to the non-migraine control group. NTG or saline was administered in blinded fashion intraperitoneally (IP) in a volume of 2 ml/kg. For Experiment 3, sumatriptan succinate (PAR Pharmaceutical, Woodcliff Lake, New Jersey, USA) was dissolved in physiological saline to doses of 0.3 and 1.0 mg/kg/ml and delivered IP.

#### Behavioral measures

All behavioral measures were collected by researchers blinded to treatment condition.

#### Rat Grimace Scale (RGS)

RGS scores were used as an index of pain severity; scoring of facial action units is described in detail elsewhere [23]. RGS has been quantified in inflammatory, neuropathic, and incision pain models but this is the first attempt to score facial action units in a model of migraine. Photos were captured using a digital camera from rats placed in 31 × 22 × 26 cm Plexiglas chambers. Photographs were randomly ordered then scored by two trained assistants; rater scores were averaged, and percent agreement was 81 % across studies.

#### Tail flick test

Warm- and cold-water tail flick tests were conducted to assess thermal allodynia. Though not an ICHD-3 criterion for migraine, allodynia was included to index sensory hypersensitivity and for comparison with prior animal studies. Water temperature in 250 ml beakers was maintained at 46 °C+/− 0.1 °C using a hot plate or at 15 °C +/− 0.1 °C using crushed ice. For testing, each rat was gently wrapped in a terry cloth towel and its tail submerged 5 cm. Latency to flick or curl the tail was recorded with a 40-s cut-off.

#### Light/dark box

A place preference apparatus (Model# MED-CPP-013, Med Associates, St. Albans, VT, USA) was modified to create a light/dark box used to quantify the ICHD-3 criteria of photophobia and reduced activity associated with migraine. Clear lids of the black and center gray chambers were covered with heavy black construction paper (inside ≤5 lux); the white chamber with clear lids served as the light portion (inside ≥635 lux). On test day, rats were placed into the center chamber for a 1 m acclimation period after which the guillotine-style doors were opened to allow access to the entire apparatus. Time in the light chamber and total number of photobeam breaks during a 10 m test session were recorded via vendor software.

#### Elevated plus maze (EPM) and forced swim test (FST)

Because frequent (ie “chronic”) migraineurs show increased rates of anxiety and depression [24–26], experiment 2 added EPM and FST assays to determine if multiple injections induced these symptoms. The EPM is a widely used assay to model generalized anxiety disorder and was used to quantify anxiety-like behavior that may be associated with repeated migraine inductions in Experiment 2. The apparatus, positioned 76 cm above the floor, consisted of four arms (56 cm), two of which have 19 cm tall walls creating the “closed” arms, while “open” arms resemble an open runway. For testing, rats were placed onto the maze center facing an open arm. Distressed rodents display increased avoidance of the open arms. During a 5 m test session, time spent in the open arms was recorded via EthoVision video tracking software (Noldus, Leesburg VA, USA).

The FST is a commonly used model of behavioral despair used to quantify depression-like behavior and was used in Exp 2 for that purpose. Rats were placed into a 20 cm diameter × 35 cm tall cylinder filled with 24-26 °C water to within 5 cm of the rim. Rats initially attempt to escape through swimming and diving; distressed rats display decreased latency to behavioral despair (ie, floating behavior). Time engaged in floating behavior, which included small movements necessary for the rat to keep its head above water, during a 5 m test session served as the dependent measure and was quantified by EthoVision video tracking software.

### Statistical analyses

Data were analyzed using SPSS with independent samples t-tests, one-way (between groups) ANOVAs, or two-way (between and within groups) ANOVAs with post-hoc tests for simple effects (Fisher’s exact or independent samples t-tests); significance was *p* < .05.

### EXPERIMENT 1: single NTG vs. Saline administration

## Procedure

Rats received either 10 mg/kg NTG or saline (*n* = 9–10 per group) IP and then were returned to their home cages. Photographs for RGS scoring were taken 60, 90, and 120 m post-injections with rats returned to their home cages between sessions. After the last photograph, rats were tested in adjacent lab on the tail flick tests (counter-balanced for order), followed by light/dark box for photophobia and locomotion.

## Results

As summarized in Table 1, NTG significantly altered only the behavioral endpoint of locomotor activity. In contrast to our prediction the NTG group exhibited more activity than the saline group, t(15) = 3.957, *p* = .022.

**Table 1:** Effects of a single NTG migraine episode on behavioral endpoints

Endpoints	Saline	NTG
Grimace Score (60-90-120 m)	.057/.025/.063 (.096/.053/.106)	.163/.207/.188 (.289/.379/.319)
Cool-Tailflick Latency (s)	16.8 (10.0)	19.2 (13.6)
Warm-Tailflick Latency (s)	8.9 (6.2)	12.3 (5.7)
Time in Light Box (s)	27.2 (12.7)	40.4 (19.6)
Locomotor Activity (# beam breaks)	105.8 (29.0)	217.8* (79.4)

Means (SD)

*p < .05 vs saline control

### Discussion

A single NTG episode produced negligent effects on photophobia, RGS, and allodynia. Increased locomotor activity of NTG rats may reflect escape-like behavior associated with ongoing pain. Noting that ICHD-3 diagnosis of migraine requires a history of multiple episodes, Experiment 2 sought to determine whether repeated NTG injections induce alterations in these behavioral endpoints and whether repeated injections induced symptoms of anxiety or depression.

### EXPERIMENT 2: repeated NTG episodes

## Procedure

Three groups of rats (*n* = 10 per group) received 5 IP injections over 2 weeks (every third day during the middle of their light cycle). Control rats received 5 saline administrations; experimental groups received either 3-NTG (alternated with 2 saline) or 5-NTG administrations. NTG doses were the same as in Experiment 1. Behavioral measures described earlier were conducted 2 h after the fifth injection in the aforementioned order. Rats were tested on the EPM the following morning and the FST that afternoon.

## Results

**Fig. 1:**
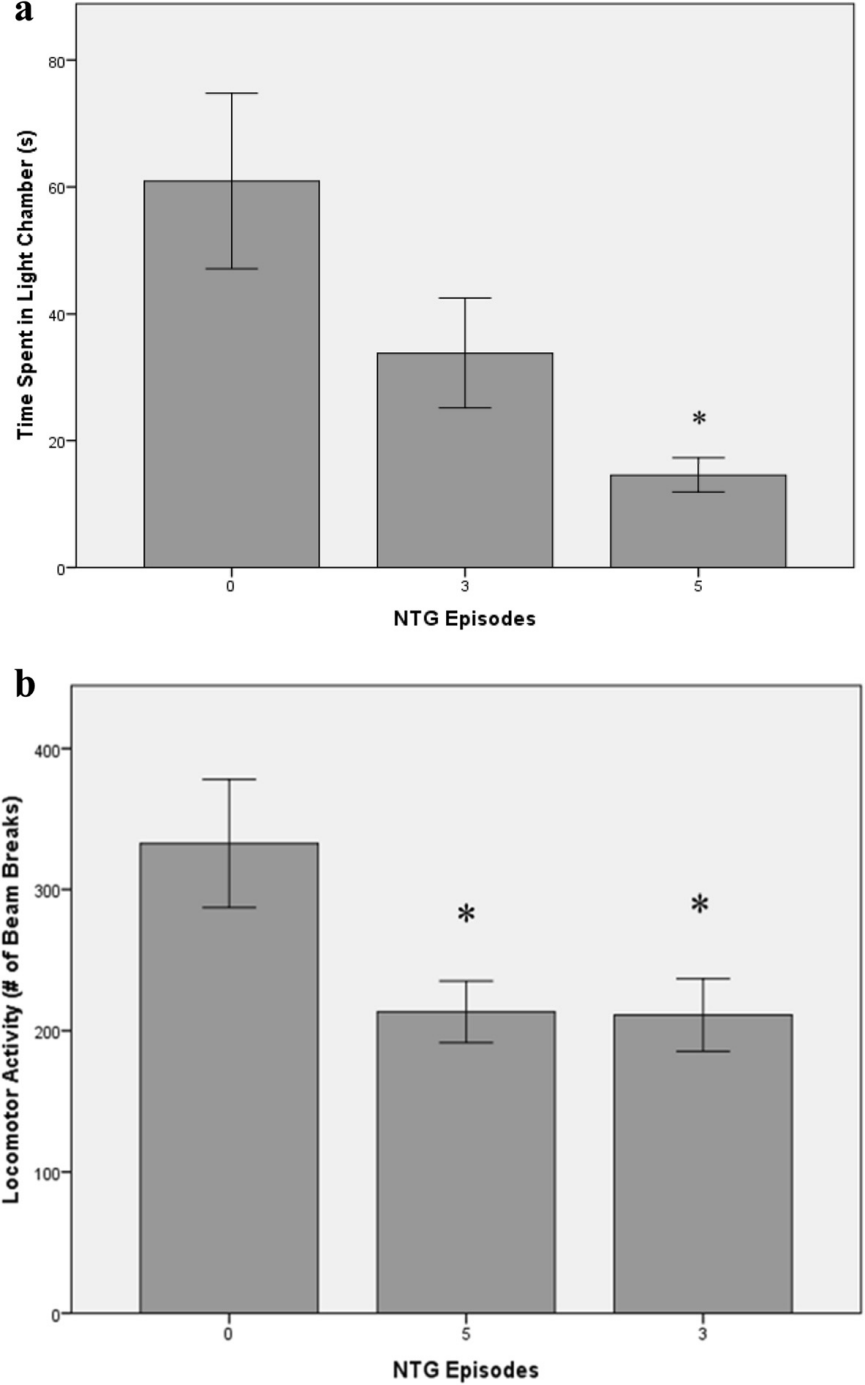
Behavioral endpoints of light sensitivity (**a**) and motor activity (**b**) as a function of NTG injection condition (*M* ± SEM) *p < .05 vs. saline control

As shown in Table 2, a main effect for body weight growth was found, F(2,27) = 6.93, *p* = .004. Planned comparisons confirmed that 3- and 5-NTG groups gained significantly less weight than controls (*p*s = .005 and .021). As before, repeated NTG did not produce significant alterations in RGS or tail-flick latencies nor affect time in the EPM or FST. As shown in Fig. 1, however, repeated administrations produced a main effect for time spent in the light portion of the light–dark box (Panel A), F(2,27) = 5.97, *p* = .007. Planned comparisons revealed the 5-NTG group spent less time in the light chamber than the 0-NTG group (*p* = .005); a similar pattern was seen in the 3-NTG group (*p* = .055). Repeated NTG episodes also yielded a main effect for locomotor activity (Panel B), F(2,27) = 4.53, *p* = .02, in which mean photobeam breaks in the 3- and 5-NTG groups were significantly lower than the 0-NTG control group (*p*s = .036 and .04, respectively).

**Table 2:** Effects of recurrent NTG migraine episodes on behavioral endpoints

Endpoints	0-NTG (5 Saline)	3-NTG (2 Saline)	5-NTG
% Change Body Weight	21.76 (3.21)	14.93* (7.49)	13.42* (4.35)
Grimace Score	.075 (.105)	.138 (.181)	.20 (.206)
Cool-Tailflick Latency (s)	16.5 (12.4)	23.0 (18.3)	20.1 (15.6)
Warm-Tailflick Latency (s)	9.8 (5.3)	10.4 (3.4)	13.2 (12.5)
EPM-Time in Open Arms (s)	13.0 (19.3)	17.4 (16.7)	16.2 (22.9)
FST-Float Time (s)	136.3 (36.1)	144.5 (41.4)	112.9 (46.0)

Mean (SD); *EPM* elevated plus maze. *FST* forced swim test

*p < .05 vs 0-NTG control

## Discussion

Repeated NTG episodes produced significant alterations in weight gain, light sensitivity and locomotor activity. Reduction in weight gain may be associated with reduced appetite that often accompanies migraine but this requires future exploration. In contrast to the aforementioned migraine-specific effects, repeated NTG administration did not produce thermal allodynia nor affect stress-related behaviors on the EPM and FST, the latter of which suggests that increased frequency of episodes may be necessary to model affective comorbidities that accompany chronic migraine. While NTG-induced thermal allodynia has been detected using the Hargreaves procedure [9], our findings suggest that this does not present to the tail.

Taken in conjunction with Experiment 1, repeated NTG administration accompanied by measures of clinically-relevant behavioral endpoints of light sensitivity and reduced locomotor activity better simulate features of human migraineurs and begin to establish the model’s validity. An additional validation step was to determine whether these endpoints are affected by sumatriptan, a common migraine abortive.

### EXPERIMENT 3: sumatriptan effects on repeated NTG episodes

## Procedure

Three groups of rats (*n* = 8–10 per group) received a series of 5 NTG injections over 2 weeks (every third day during the middle third of their light cycle). Thirty minutes after each administration, rats received either saline or 0.3 or 1.0 mg/kg sumatriptan IP and were returned to their home cages. RGS and light/dark box assays were conducted 90 m after the fifth administration. FST and EPM assays were not included in Experiment 3 because evidence across behavioral assays in Experiment 2 confirmed the utility of the 5-NTG protocol in modeling episodic but not chronic migraine nor its associated affective comorbidities.

## Results

**Fig. 2:**
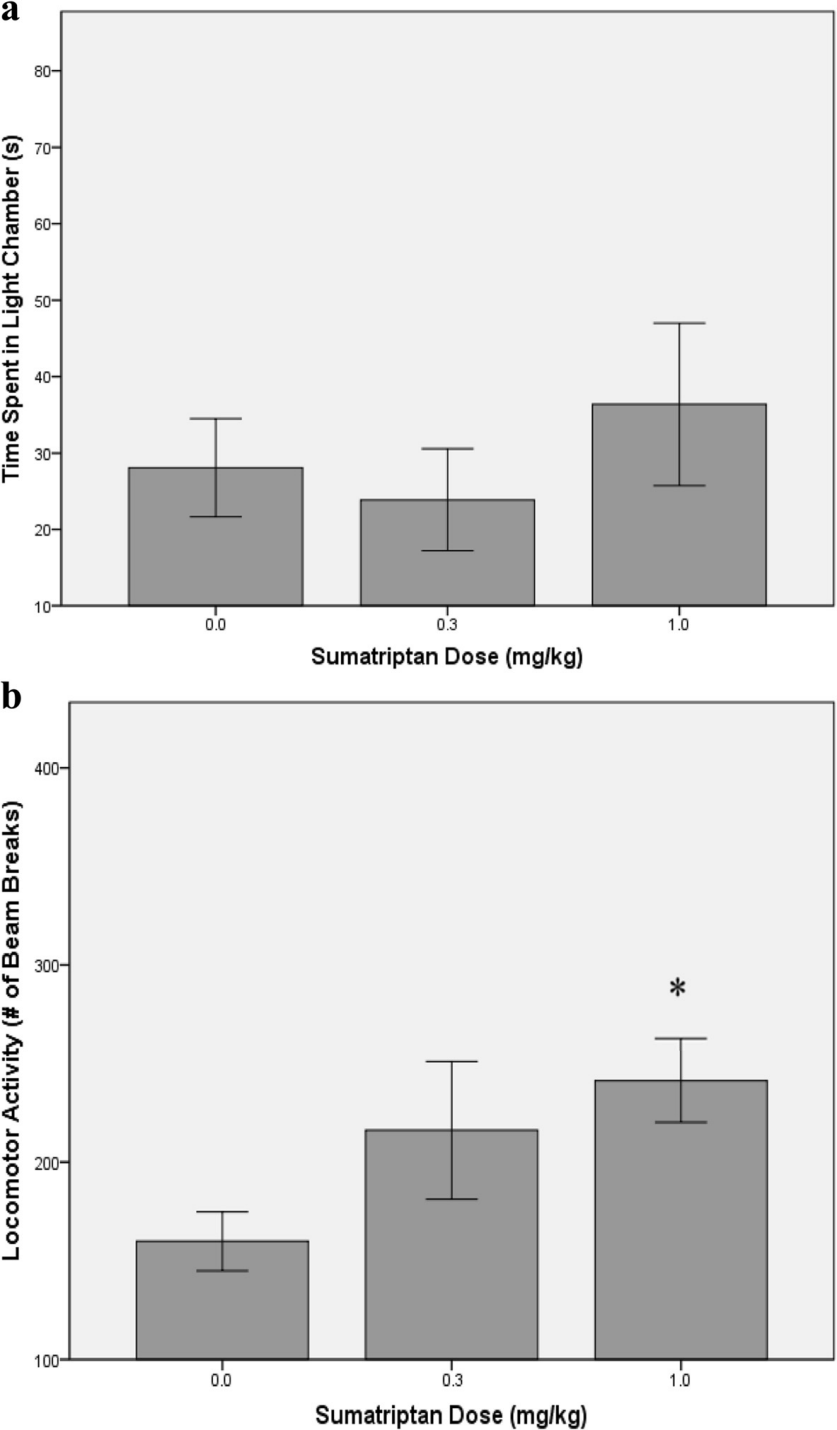
Behavioral endpoints of light sensitivity (**a**) and motor activity (**b**) as a function of sumatriptan condition (*M* ± SEM) *p < .05 vs. saline control

Though sumatriptan had modest effects on body weight change, a significant main effect was found for RGS, F(2,24) = 6.23, *p* = .007, such that the mean RGS scores for both sumatriptan groups were significantly lower than the saline group (*p*s < .006), confirming that sumatriptan reduced pain intensity (see Table 3). Sumatriptan did not significantly affect photophobia as all rats spent little time in the light portion of the box (Fig. 2, Panel a) but dose-dependently attenuated reductions in locomotor activity in a manner that approached significance, F(2,24) = 2.40, *p* = 0.112. Planned comparisons revealed that animals treated with 1.0 mg/kg sumatriptan had significantly more photobeam breaks (increased activity) than the saline group (*p* < 0.045).

**Table 3:** Effects of sumatriptan on body weight and grimace scores after five NTG episodes

Endpoints	Saline	Sumatriptan 0.3 mg/kg	Sumatriptan 1.0 mg/kg
% Change Body Weight	−3.03 (4.11)	−0.06 (4.81)	1.84* (4.33)
Grimace Score	.55 (.50)	.14^a^ (.14)	.09* (.11)

Mean (SD)

*p < .05 vs saline control

## Discussion

Results demonstrate that at least one of the clinically-relevant endpoints of repeated NTG-migraine can be attenuated by sumatriptan (i.e., hypoactivity). The inability to reverse alterations in light sensitivity may be related to testing in a novel environment after only the final NTG episode, and exposure to the box after each injection might afford detection of more robust drug effects. Interestingly, sumatriptan attenuated RGS scores and alterations in body weight associated with repeated NTG episodes. Differences in body weight changes between Experiments 2 and 3 likely reflect differing initial body weights due to limits in animal availability from the vendor: Experiment 2 used younger rats within their main growth curve function while Exp 3 used older rats nearer their body weight plateau. Whether body weight changes reflect an indirect measure of nausea or reduced appetite remains to be determined. We found quantification of RGS to be challenging as the observed alterations in facial action units are not as robust as seen in other pain models (mean RGS scores >1.0 are found in neuropathic and arthritic models), and thus believe RGS is unlikely to be a suitable behavioral endpoint for modeling migraine.

## Conclusions

The overall aim of these three studies was to further validate an NTG migraine model in rodents by employing an endophenotypic mapping strategy that asked two questions related to establishing homologies in symptoms and pharmacological response: First, do single or multiple NTG injections produce behavioral endpoints that simulate the clinical presentation of spontaneously-emitted migraine symptoms in humans? Second, does sumatriptan attenuate these endpoints in rats as it does in humans? Multiple injections were used to mirror the minimum headache attacks necessary for diagnosis [14] and behavioral endpoints chosen that mapped onto ICHD-3 diagnostic criteria (e.g., photophobia, aggravation by activity).

Our results indicate that recurrent NTG episodes are necessary to affect clinically-relevant symptoms of light sensitivity and activity in a rat migraine model. Other laboratories have reported that multiple NTG episodes may model processes of migraine progression [15], and our findings add behavioral endpoints to strengthen this clinical simulation. Finally, sumatriptan administration successfully modulated activity but not photophobia in the light–dark box; this may be attributable to an absence of exposure to the Fight–dark box during prior NTG episodes preventing avoidance learning.

Current research in our lab is exploring protocol variations that may increase effects on the clinically-relevant behavioral endpoints. Once accomplished, several lines of research may further validate this recurrent NTG migraine model. For example, this model should be sensitive to established migraine preventive pharmacotherapies [15], as well as novel abortive treatments such as those targeting cannabinoid receptors [27]. Utilization of more frequent NTG injections over longer time periods may better model the phenomenon of chronic migraine and associated states of allodynia and comorbid psychiatric symptomatology [18, 26]. Another promising line of research would be to determine whether these endpoints are influenced by estrous cycle stage or manifest in other models of migraine induction [10, 11]. Collectively, the work described herein and that of other researchers in modeling migraine will ultimately provide better simulations with greater translational value, leading to further insights into both the pathophysiology and treatment of this common and debilitating condition.
